# A new material platform of Si photonics for implementing architecture of dense wavelength division multiplexing on Si bulk wafer

**DOI:** 10.1080/14686996.2017.1301193

**Published:** 2017-04-13

**Authors:** Ziyi Zhang, Motoki Yako, Kan Ju, Naoyuki Kawai, Papichaya Chaisakul, Tai Tsuchizawa, Makoto Hikita, Koji Yamada, Yasuhiko Ishikawa, Kazumi Wada

**Affiliations:** ^a^ Department of Materials Engineering, University of Tokyo, Bunkyo, Japan; ^b^ Nanophotonics Center and NTT Device Technology Laboratories, Nippon Telegraph and Telephone Corporation, Atsugi, Japan; ^c^ NTT-Advanced Technology, Mitaka, Japan; ^d^ Massachusetts Institute of Technology, Cambridge, MA, USA

**Keywords:** Integrated optics, integrated photonics, Si photonics, WDM on a chip, mid-index contract optics

## Abstract

A new materials group to implement dense wavelength division multiplexing (DWDM) in Si photonics is proposed. A large thermo-optic (TO) coefficient of Si malfunctions multiplexer/demultiplexer (MUX/DEMUX) on a chip under thermal fluctuation, and thus DWDM implementation, has been one of the most challenging targets in Si photonics. The present study specifies an optical materials group for DWDM by a systematic survey of their TO coefficients and refractive indices. The group is classified as mid-index contrast optics (MiDex) materials, and non-stoichiometric silicon nitride (SiN_x_) is chosen to demonstrate its significant thermal stability. The TO coefficient of non-stoichiometric SiN_x_ is precisely measured in the temperature range 24–76 °C using the SiN_x_ rings prepared by two methods: chemical vapor deposition (CVD) and physical vapor deposition (PVD). The CVD-SiN_x_ ring reveals nearly the same TO coefficient reported for stoichiometric CVD-Si_3_N_4_, while the value for the PVD-SiN_x_ ring is slightly higher. Both SiN_x_ rings lock their resonance frequencies within 100 GHz in this temperature range. Since CVD-SiN_x_ needs a high temperature annealing to reduce N–H bond absorption, it is concluded that PVD-SiN_x_ is suited as a MiDex material introduced in the CMOS back-end-of-line. Further stabilization is required, considering the crosstalk between two channels; a ‘silicone’ polymer is employed to compensate for the temperature fluctuation using its negative TO coefficient, called athermalization. This demonstrates that the resonance of these SiN_x_ rings is locked within 50 GHz at the same temperature range in the wavelength range 1460–1620 nm (the so-called S, C, and L bands in optical fiber communication networks). A further survey on the MiDex materials strongly suggests that Al_2_O_3_, Ga_2_O_3_ Ta_2_O_5_, HfO_2_ and their alloys should provide even more stable platforms for DWDM implementation in MiDex photonics. It is discussed that the MiDex photonics will find various applications such as medical and environmental sensing and in-vehicle data-communication.

## Graphical abstract

**Figure:**
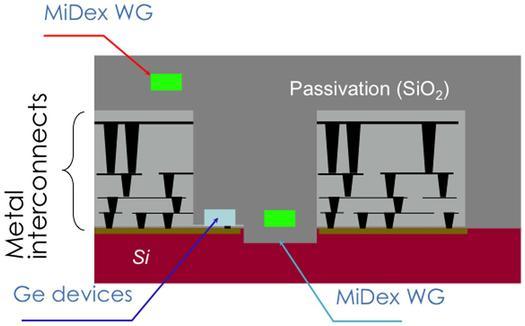

## 1. Introduction

**Figure 1.**
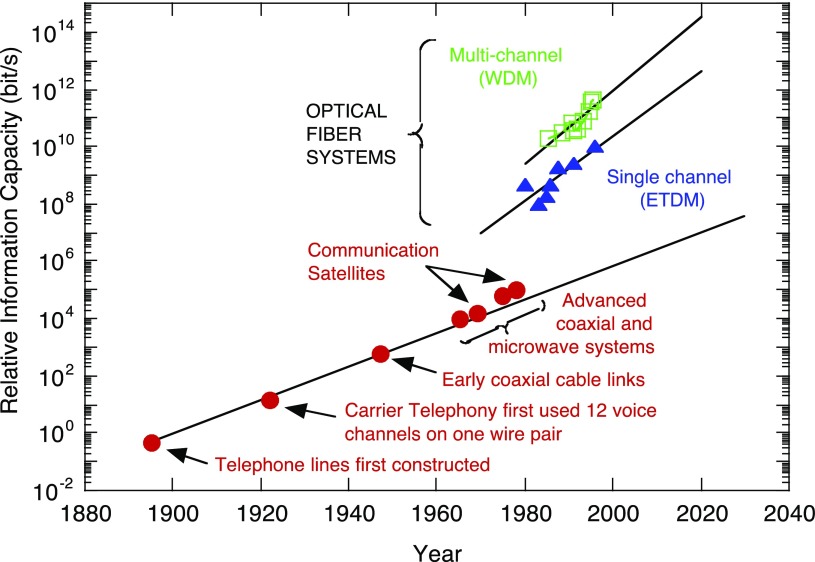
Information capacity vs. year. Signal multiplexing technologies, such as wavelength division multiplexing (WDM) and electrical time division multiplexing (ETDM), assume the most vital role to meet the considerable growth of communication capacity. Reproduced with permission from *The Electrochemical Society Interface*, 9–2, 20, (2000).

**Figure 2.**
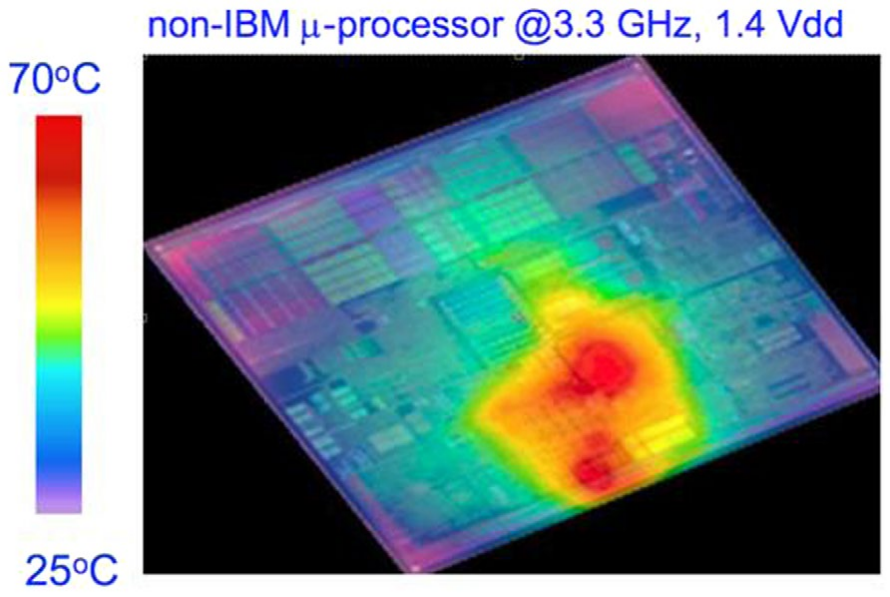
Local heating of central processing unit (CPU). This figure is from [9], which is in black and white. Thanks to Y. Vlasov for the color version.[10]

Si photonics enables cost-effective wide-bandwidth communication and computation networks, and will shortly appear in data centers and in our daily life [1–7]. The figure of merit (FoM) of a network system is given by 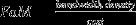. The question of why Si photonics is preferable to Si electronics can be answered by considering optical fiber communication. Figure 1 shows progress in information capacity [8]. Starting with voice communication between Bell and Watson through their telephone demonstration between Boston and Cambridge in 1875, there was exponential increase in information capacity by five orders of magnitude in nearly 100 years due to ‘electrical’ communication (red circles). There is a large jump in 1980 brought about by optical fiber communication. This technology instantaneously increased information capacity by three orders of magnitude, much faster than was possible by the previous technology. The green squares in Figure 1 signify that 100 colors of light were used to communicate (green squares), which increased the information capacity by 100 times compared with using one color (blue triangles). Wavelength division multiplexing (WDM) is very important in optical communication. The fundamental device is a prism filter besides light emitters and detector, which is called multiplexing/demultiplexing (MUX/DEMUX). The International Telecommunication Union – Telecommunication Standardization Sector (ITU-T) recommends the following protocol for WDM: the difference in wavelength between colors of light, i.e. channel spacing, should be 100 GHz (0.8 nm), with a crosstalk lower than –30 dB in the wavelength range near 1.55 μm. This is referred to as dense WDM (DWDM). This is the requirement to MUX/DEMUX as well as light emitter. Despite its significant advantage, implementation of the DWDM architecture in Si photonics remains a ‘holy grail’. This is because Si has a large thermo-optic (TO) coefficient and MUX/DEMUX is extremely sensitive to the ambient temperature. On the other hand, Si complementary metal oxide large-scale integrated (CMOS LSI) circuitry is based on ‘uncooled chip’ architecture, and works with no thermal management. Then the chip is locally heated up to ~70 °Cas in Figure 2 [9,10]. DWDM thus malfunctions due to temperature fluctuation when integrated on a Si chip.

The present paper reviews the conflict of on-chip DWDM and uncooled architectures, summarizes our survey on TO coefficients and bandgaps of various optical materials, and proposes a materials group of mid-index contrast optics (MiDex) materials for DWDM on a chip. The main results are as follows:

(1) Non-stoichiometric silicon nitrides (SiN_x_) are chosen as a typical MiDex material from the design figure and prepared by physical vapor deposition (PVD) and chemical vapor deposition (CVD) methods.
(2) These TO coefficients are precisely measured. The ring resonators are chosen as MUX/DEMUXs in the present work and prepared by PVD- and CVD-SiN_x_. These rings can lock the resonance frequency within 100 GHz in the temperature range 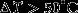, while the temperature range for Si is only 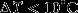.
(3) The PVD-SiN_x_ platform can be integrated in the CMOS back-end-of-line because there is no need for high temperature annealing to reduce N–H bond associated absorption at around 1520 nm. To fully meet the ITU-T protocol, the crosstalk of –30 dB, the channel spacing has to increase by 32 GHz in the present rings of Q of 31,600. Thus, the channel spacing must be 132 GHz, suggesting that further stabilization is required.
(4) Athermalization of the ring with a ‘silicone’ polymer shrinks the channel spacing to 50 GHz in the wavelength range from 1460 nm to 1620 nm (the so-called S, C, and L bands) for 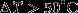. Thus, ITU-T protocol is met because the channel spacing with the crosstalk is 82 GHz, i.e. <100 GHz.
(5) An extensive survey of MiDex materials, including materials with high dielectric constants (*k*), i.e. high *k* materials reported in Si-LSIs studies, is performed. It is proposed from the revised design figure that Al_2_O_3_, Ta_2_O_5_, Ga_2_O_3_, HfO_2_ and their alloys should be the choice of MiDex materials for on-chip DWDM implementation without athermalization.

MiDex-based electronic and photonic integrated circuits (EPICs) for DWDM are illustrated on a bulk Si wafer. In MiDex there is no need for a SOI wafer. The potential applications of the MiDex photonics are discussed, such as medical and environmental sensing, as well as data communication in vehicles and data centers.

## 2. Minimum channel spacing

**Figure 3.**
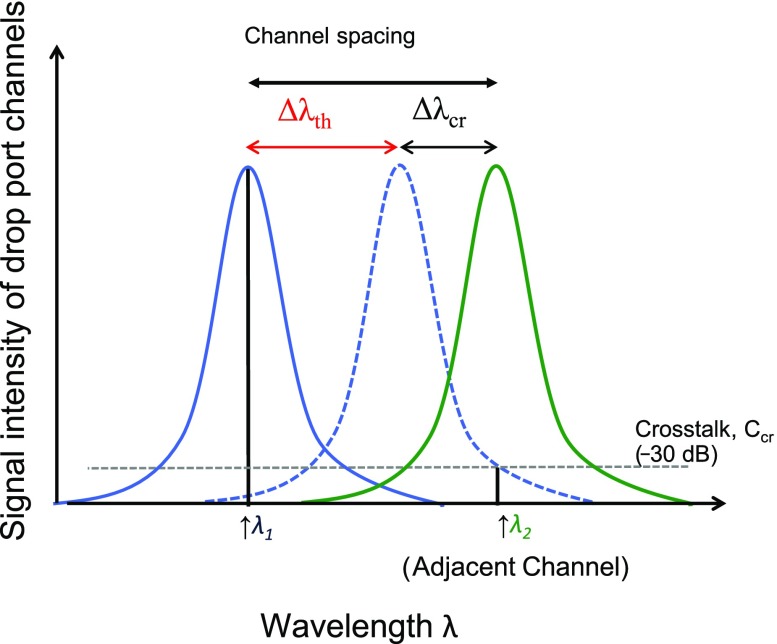
Minimum channel spacing. Thermal shift and crosstalk should determine the minimum channel spacing.

The impact of temperature fluctuation on DWDM is explained in this section. Here, a ring resonator is used as MUX/DEMUX. Figure 3 illustrates the relation between the filtering characteristics of the ring and the minimum channel spacing 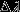. It is assumed that the filtering wavelength is originally at 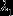. The thermal shift amount of the wavelength 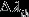 can be described by the EO coefficient of the ring material since 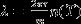, where r denotes the ring radius, m integer multiple, and n(T) effective refractive index of the ring material. The linear-approximation can be 
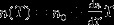
. Thus, 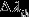 can be given by these equations. The wavelength 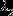 of the adjacent channel should fulfill 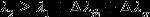 to prevent MUX/DEMUX from malfunctioning. In other words, the minimum channel spacing 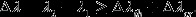 Here, 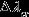 is the crosstalk and given by:[11](1)
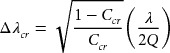

(2)
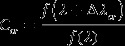



The filter spectrum 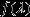 is written by the Lorenz function and Q denotes the quality factor of the ring. Accordingly, to fulfill the ITU-T recommendation 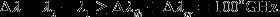 Table 1 shows the crosstalk as a function of Q at 1550 nm. The crosstalk recommended by ITU-T is –30 dB. Assuming no thermal shift, the minimum channel spacing should be 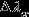; 107 GHz when Q is 10,000, and 10.7 GHz when Q is 100,000. We have also added the case of –20 dB crosstalk in Table 1, as assumed in [12]. The crosstalk of –20 dB seems beneficial in the minimum channel spacing, but the effect is not too large. This, together with the large TO coefficient of Si shown later, clearly indicates that the ITU-T recommendation is very difficult for Si photonics to meet. Therefore, it is important to find a material group of a small TO coefficient and with the ring of high Q for MUX/DEMUX. The goal of the present study is to find the material group of small TO coefficients for Si photonics to implement on-chip DWDM.

**Table 1.** The relation of minimum channel spacing with Q factor at 1550 NM.

Quality factor (Q)	Minimum channel spacing (GHz)	
	Crosstalk of −30 dB	Crosstalk of −20 dB
10^4^	107	60
3 × 10^4^	36	20
10^5^	10.7	6

Note:

Here, no thermal shift is assumed.

## 3. MiDex Si photonics platform

**Figure 4.**
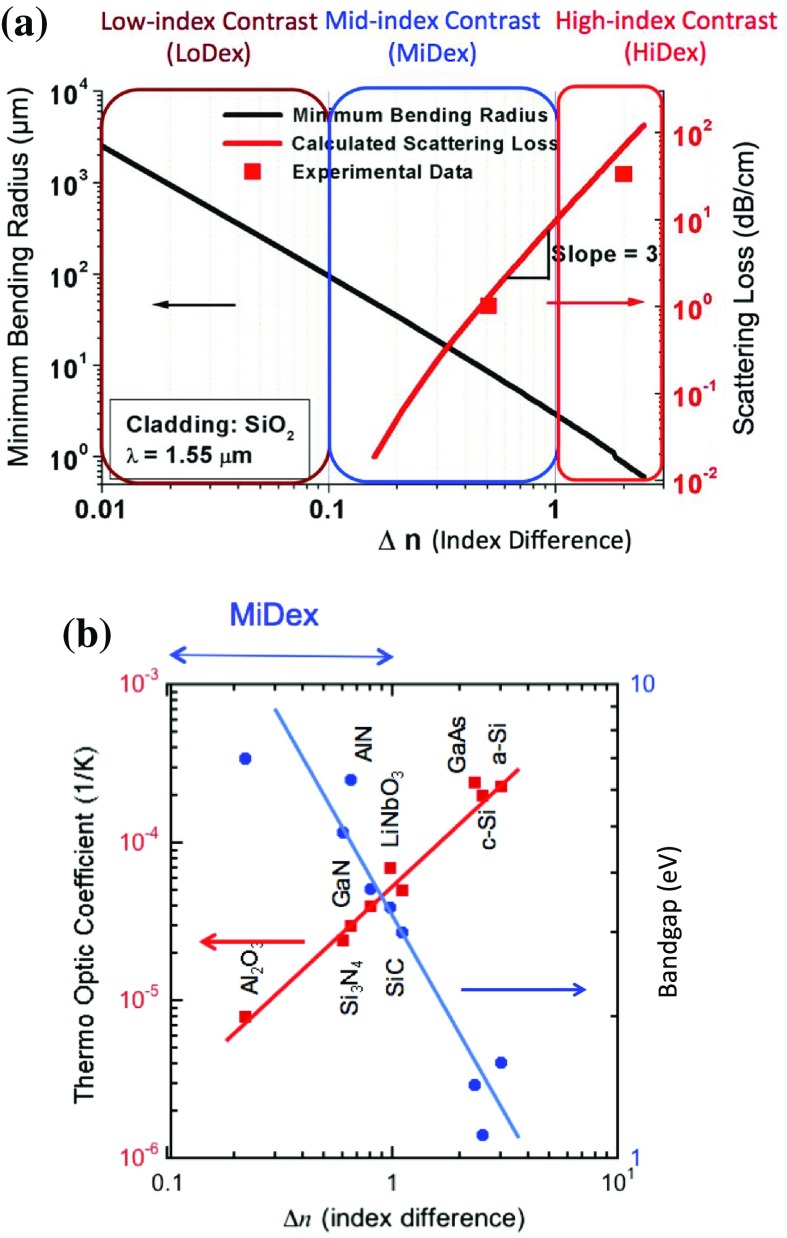
Materials chart for Si photonics platform. MiDex and HiDex are shown. Horizontal axis shows the optical index difference, Δ*n* = *n*_core_ – *n*_clad_ at 1.55 μm, where the cladding is SiO_2_. (a) Design figure for minimum bending radius and scattering loss with experimental results shown by squares. (b) Design figure for thermo-optic coefficient and bandgap of materials group. Symbols show literature data. Calculation of minimum bending radius is regarding that bending loss less than 0.1 dB/turn as acceptable.

Figure 4(a) shows the design of the minimum bending radius and scattering loss of waveguide vs. index difference (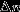) between waveguide core and cladding [1]. Here, SiO_2_ is assumed as the cladding material. Si photonics has been catalyzed by its high 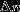, allowing a small minimum-bending radius of the waveguides, e.g. 1 μm, and thus requiring only a small footprint in integrated photonics. The index regime has been referred to as high-index contract optics by Haus [11]. Following him, we referred it as a HiDex platform. Although a high scattering loss associated with high 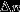 was disadvantageous, high precision fabrication technologies developed for the CMOS fabrication has solved this drawback. In contrast, low-index contrast optics (LoDex) platform based on SiO_2_ fiber technology is advantageous in the high transmission at the cost of large minimum bending radii of the waveguide (>cm). There is a regime between HiDex and LoDex, 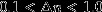, here referred to as MiDex (mid-index contrast optics). The advantage of the regime as the platform is shown in Figure 4(b) with their TO coefficients and bandgaps vs. 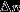 [13–16]. It is clearly seen that crystal Si (c-Si) as well as amorphous Si (a-Si) has a larger TO coefficient than the MiDex materials, here LiNbO_3_, AlN, stoichiometric silicon nitride (Si_3_N_4_), and Al_2_O_3_. This strongly suggests that the MiDex platform should be more robust to thermal fluctuation than HiDex platforms.

A small TO coefficient is a necessary condition for DWDM implementation in Si photonics. The sufficient condition is a large bandgap. Since implementing many wavelength channels in DWDM increases the overall optical power transmitting in the Si waveguide, light is absorbed through optical nonlinear phenomena in Si. The light energy of 1550 nm is 0.8 eV, and the bandgap of Si is 1.12 eV at room temperature. Thus, two photon absorption and four wave mixing happen in the Si waveguide with an increase in the channel number. To avoid these nonlinear phenomena, we should set the minimum bandgap of waveguide materials, here 3 eV, which is more than three times larger than the light energy. It is clearly shown from Figure 4(b) that the MiDex materials have all cleared the sufficient condition as well.

Among these MiDex materials frequently used in electronic and photonics, the present paper has chosen Si_3_N_4_ because it is commonly used in Si CMOS chips. Si_3_N_4_ has a TO coefficient of 2.4 × 10^−5^ 1/K which is ~1/10 that of Si, and a bandgap of ~5 eV, which is ~5 times larger than that of Si. It should thus be an excellent example to understand how MUX/DEMUX of the MiDex material behaves under thermal fluctuation. Figure 4(a) also shows that Si_3_N_4_ on SiO_2_, i.e. 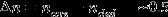, allows ~4 μm as the minimum bending radius, which is larger than that of Si but still acceptable on a Si chip of 1 × 1 cm^2^. The scattering loss is ~1 dB cm^–1^, which can be further reduced by high precision fabrication.

The drawback is that CVD-Si_3_N_4_ has N–H (nitrogen and hydrogen) bonds that induce light absorption at 1520 nm, and removing the bonds needs high temperature annealing, e.g. at 1100 °C. This prevents the Si_3_N_4_ platform from being implemented in the CMOS back-end-of-line. We will demonstrate that the PVD method solves the drawback as in Section 5.

There have been a large number of reports on this material set, stoichiometric Si_3_N_4_ on SiO_2_ to apply the waveguides. One of the earliest papers of its waveguide application was published in 1977 by Stutius and Streifer [16], a decade earlier than the Si photonics proposal [17]. They demonstrated its excellent characteristics as a waveguide core material at a visible wavelength (0.633 μm). Since then various fabrication technologies have been employed to deposit silicon nitrides on SiO_2_ to fabricate waveguides and resonators. Typical reports are on CVD [18–27] and on PVD [28–31]. Even optical transceivers based on silicon nitrides have been reported recently [12,32,33]. However, there only a few publications on measurements of the TO coefficients of silicon nitrides on SiO_2_ [15,25]. In [25], PVD-based non-stoichiometric silicon nitride (SiN_x_) films were deposited to fabricate a photonic crystal structure to measure the TO coefficient. The TO coefficient reported was 0.47 × 10^−4^ 1/K at 1510 nm, which is twice higher than that reported in [15] where a ring resonator was used but the material is stoichiometric Si_3_N_4_ and the fabrication method is CVD. Since stoichiometric Si_3_N_4_ has large built-in stress preventing a thick structure like waveguides, a clear demand exists for measurement of the TO coefficients of less stressed (thick enough) non-stoichiometric SiN_x_ fabricated by both CVD and PVD at low temperature.

## 4. Fabrications

We used radio frequency (RF) reactive sputtering to deposit PVD-SiN_x_ films on SiO_2_ thermally grown on Si. Here, Ar plasma with a Si target and ambient N_2_ were used. The substrate was kept at room temperature and the chamber pressure was controlled to be 0.5 Pa during the deposition process. SiN_x_ films were also prepared using electron cyclotron resonance plasma enhanced CVD (ECR-CVD), which allows low temperature deposition and less stressed SiN_x_. The condition was 200 °Con the SiO_2_ on Si, using SiH_4_ and N_2_. However, even ECR-CVD-SiN_x_ (CVD-SiN_x_ hereafter) was limited to ~650 nm in thickness before showing cracks on the surface. Although the PVD-SiN_x_ films can be deposited beyond 800 nm in thickness, 650 nm thick PVD-SiNx was chosen to deposit to compare the film qualities of CVD-SiN_x_. The refractive indices (*n*) of the as-deposited PVD- and CVD-SiN_x_ films were measured to be 1.96 and 1.98 at 1551 nm using spectroscopic ellipsometry; *n* values of the films were slightly smaller than that of stoichiometric Si_3_N_4_ reported as 2. To reduce N–H bond-related absorption, high temperature annealing was performed on the CVD-SiN_x_ film at two temperatures 900 and 1150 °Cfor 3 h in N_2_ gas flow. It should be noted here that the PVD-SiN_x_ film was not annealed in the present paper. Finally, single mode waveguides and ring resonators were fabricated on the PVD-SiN_x_ and on two kinds of the CVD-SiN_x_ films with and without the annealing. We used an inductive coupled plasma reactive ion etching (ICP-RIE) with CHF_3_ gas. One mask set was used to ensure identical device structures on these PVD- and CVD-SiN_x_ films. Typical waveguide structures were 650 nm thick and 1 μm wide SiN_x_ on 15 μm-thick SiO_2_ on Si for single-mode propagation. The waveguide was 5 mm long. The transmission loss was measured by the cutback method. The ring resonator was 60 μm in radius and the gap between the ring and the waveguide was 400 nm wide. The upper cladding was air. The resonance peaks of these rings were measured at temperatures from room temperature (24 °C) to 76 °Cto measure the TO coefficients of these PVD- and CVD-SiN_x_ films. For temperature calibration of the heating unit, a Si ring resonator fully covered with SiO_2_ cladding was prepared, considering the mode confinement factor. Athermalization to further stabilize the wavelength channel was studied using a ‘silicone’ polymer as the upper cladding.

## 5. Results and analysis

### 5.1. Waveguides and ring resonators

**Figure 5.**
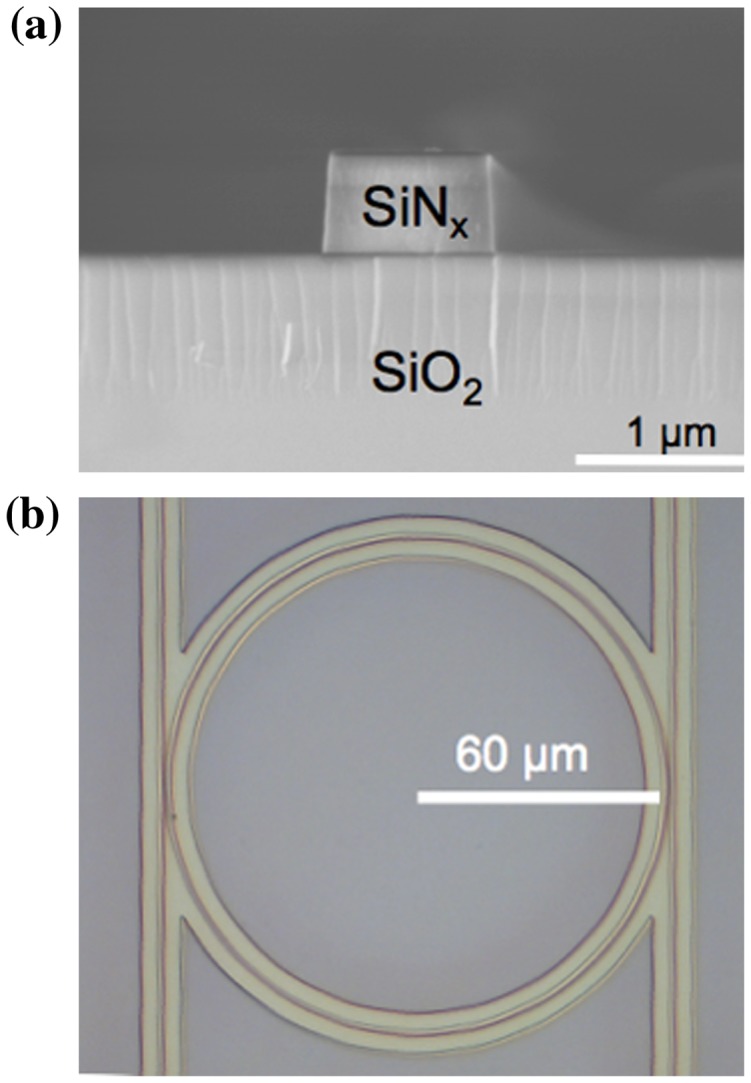
Typical SEM images of fabricated SiN_x_. (a) A cross sectional SEM image, (b) a plan view image of SiN_x_ ring and coupled waveguides used in the present work.

Figure 5 shows scanning electron microscopy (SEM) images of typical waveguides and coupled ring resonators fabricated using the PVD method [13]. The waveguides and ring resonators are fabricated as designed. No cracks were observed on PVD- and CVD-SiN_x_, indicating their small built-in stress in the present non-stoichiometric SiN_x_.

### 5.2. N–H bond related absorption

**Figure 6.**
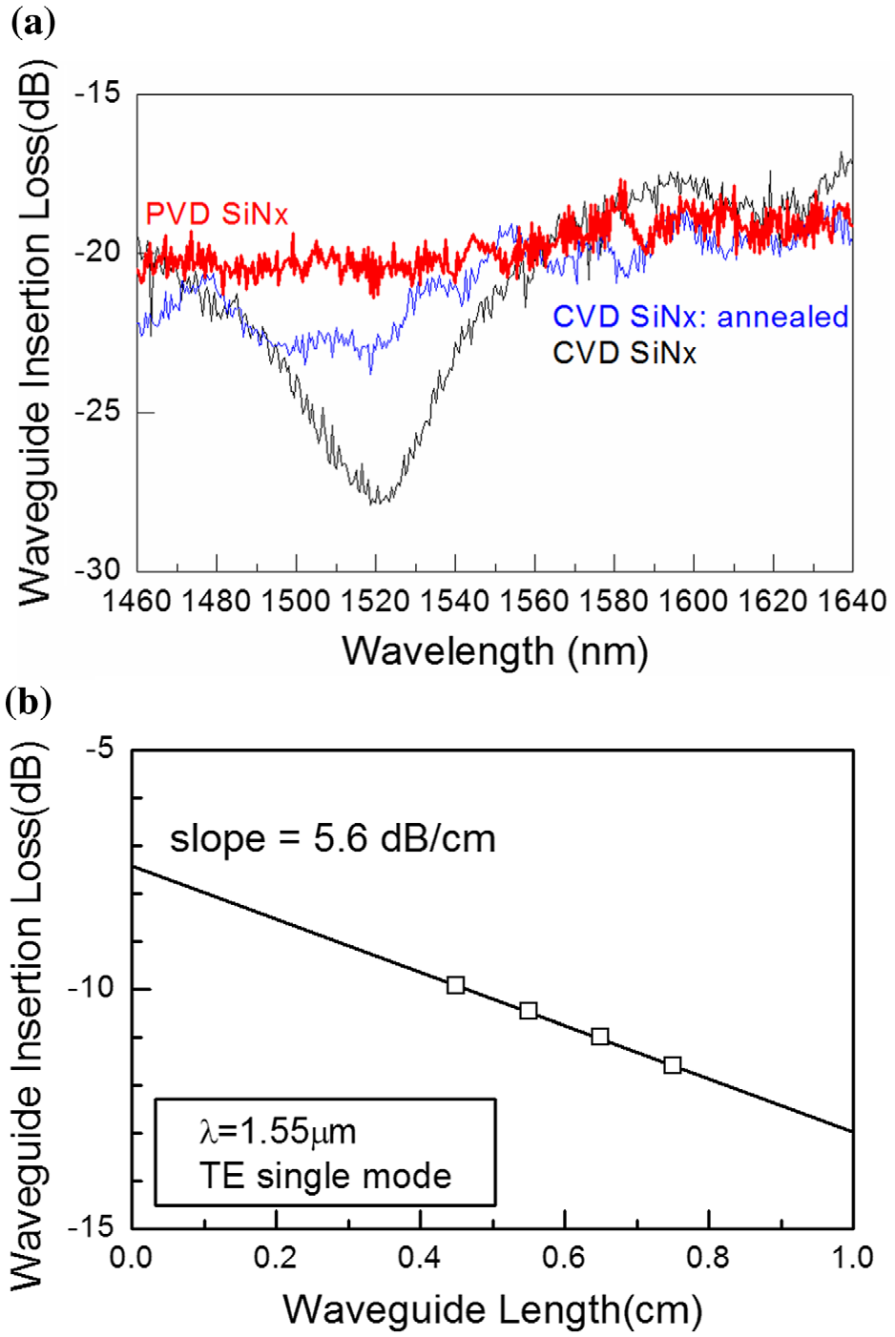
Transmission characteristics of PVD- and CVD- SiN_x_ waveguides on SiO_2_. (a) Transmission spectra. The PVD-SiN_x_ waveguide is shown in red, the CVD-SiN_x_ is plotted in black and in blue (annealed at 900 °Cfor 3 h). The transmission loss of the CVD-SiN_x_ after 1150 °Cannealing for 3 h (not shown here) is identical to that of the PVD-SiN_x_ (red). (b) The transmission loss of the PVD-SiN_x_ waveguide. The loss ~5.6 dB cm^–1^ is obtained using the waveguide with air upper cladding. Simulation indicates that the loss is reduced to ~1 dB cm^–1^ with the SiO_2_ upper cladding.

Figure 6 shows transmission characteristics of the PVD- and CVD-SiN_x_ waveguides [13]. It is clearly shown in Figure 6(a) that the PVD-SiN_x_ waveguide (red) has no absorption peak, while the CVD-SiN_x_ waveguide (black) without annealing has a broad absorption peak centered at 1520 nm. The peak still remains after the sample is annealed at 900 °Cfor 3 h (blue) but disappears by annealing at 1150 °Cfor 3 h and is similar to that of the PVD-SiN_x_. Therefore, it is fair to conclude that the absorption is generated by N–H bonds in the CVD-SiN_x_ film, and that the absorption is not detected in the PVD-SiN_x_ film. The PVD process does not use any hydrogen, and thus no absorption at 1520 nm is quite reasonable.

Figure 6(b) shows that the transmission loss of the PVD-SiN_x_ waveguide is ~5.6 dB cm^–1^ at 1550 nm for the transverse electric (TE) mode. Finite difference time domain simulation indicated the transmission loss of the waveguide with air cladding would be associated with the sidewall scattering of the waveguide, and should reduce to ~1 dB cm^–1^ by a SiO_2_ upper cladding of the waveguide. This is the same as previously reported in CVD-SiN_x_ [21,22]. Therefore, the present PVD-SiN_x_ waveguide is as transparent as the CVD-SiN_x_ waveguide annealed at a high temperature.

The transmission loss in our waveguide is much lower than in previous reports on PVD-SiNx [28–31]; tens of dB cm^–1^ even after 1050 °Cannealing for 2 h [31]. There are at least two differences from the present study: (i) their *n* reported was ~2.2, while ours was 1.96; (ii) their PVD-SiN_x_ was deposited using a SiN_x_ target while ours used a Si target with nitrogen plasma. The difference in *n* strongly suggests that their PVD-SiN_x_ would be Si-rich, which may result in the high transmission loss in terms of scattering. However, *n* of 2.2 was also reported in CVD-SiN_x_ waveguides by other researchers [19], showing the transmission loss was only 2.1 dB cm^–1^. The difference between [31] and the present work still exists in the SiN_x_ target. Assuming that the target was prepared by CVD and contains a high density of N–H bonds, the low transparency can be understood. However, their loss does not decrease even after 1050 °Cannealing for 2 h, which should be enough high to anneal hydrogen out. Thus, it is fair to say that the origin of the high transmission loss reported remains unclear.

To summarize the results we obtained here:

(1) The waveguides fabricated from as-deposited PVD-SiN_x_ film are as transparent as the ones from CVD-SiN_x_ annealed at a high temperature to remove N–H bonds.
(2) The transmission loss of the PVD-SiN_x_ waveguide is ~5.6 dB cm^–1^ with an air-cladding. Simulations suggest it reduces to ~1 dB cm^–1^ with a SiO_2_ upper cladding, which is the same as the reported results on CVD-SiN_x_ after the high temperature annealing.

In addition, PVD SiN_x_ is advantageous over CVD-SiN_x_ in the maximum thickness of deposited layers, due to its lower built-in stress. This is beneficial to minimize so called polarization depend loss.

### 5.3. Measurement of thermo-optic coefficient

In DWDM implementation it is required that the channel wavelength of MUX/DEMUX should be unchanged under thermal fluctuation. The thermal shift in resonance peaks of ring resonators can be expressed as:(3)
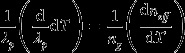



where *λ*_*p*_ is the resonance wavelength peak, and *n*_*g*_ and *n*_*eff*_ are group index and effective index, respectively. Equation (3) does not take into account the effects of the wavelength dispersion of the TO coefficient, of the second order terms and of the thermal expansion coefficient. The right side of Equation (3) is obtained by solving the propagation mode shape in the present waveguides with the refractive index profile. The refractive indices and TO coefficients 1/K of Si, CVD-SiN_x,_ and SiO_2_ at ~1550 nm were 18.0 × 10^−5^ [34,35], 2.45 × 10^−5^ [14], and 0.85 × 10^−5^ [14].

**Figure 7.**
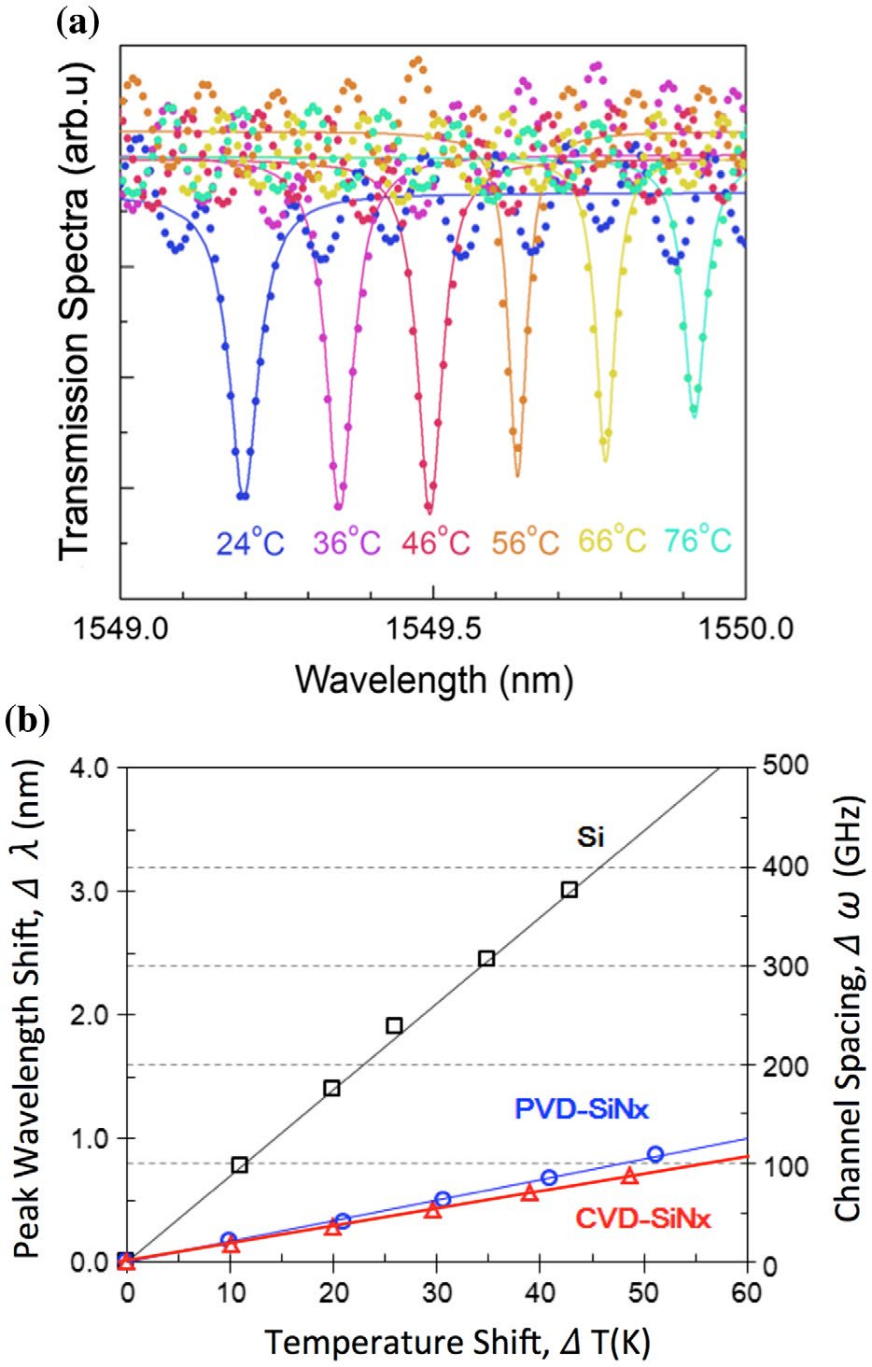
Transmission characteristics of the PVD- and CVD-SiN_x_ ring resonators at temperatures from room temperature (24 °C) to 76 °C. (a) The spectra of the drop port show Q of 31,600. (b) The resonance peak shifts of CVD-SiN_x_ rings are reproduced by the reported thermo-optic coefficient, 2.4 × 10^−5^ 1/K, obtained for CVD stoichiometric Si_3_N_4_.[15] The shifts are slightly larger for PVD than CVD rings. The thermo-optic coefficient of the PVD-SiN_x_ is 2.8 × 10^−5^ 1/K.

Figure 7 shows the measured peaks in transmission spectra with Lorentzian fittings [13]. The quality factor Q of these peaks is calculated to be 31,600. Measured peak shifts are 20.4 and 16.2 pm K^–1^ for the PVD- (blue) and CVD-SiN_x_ (red) rings. The CVD-SiN_x_ result agrees extremely well with a previous report [15], which indicates that the TO coefficient of CVD-SiN_x_ is almost same as the reported stoichiometric Si_3_N_4_, but a half of that report [25]. In [25], photonic crystal structures of multilayer stacks were used to measure the TO coefficient, while the present study and the report [13] use thin layer structure of ring resonators. It may be fair to say that the origin of the TO coefficient difference from [25] would be the multilayer stack structure of their photonic crystal, most likely stress induced by the stack structures. On the other hand, the PVD-SiN_x_ results show a slightly larger TO coefficient, i.e. 2.8 × 10^−5^ 1/K, than CVD-one. The origin of the difference is not clear.

In conclusion, the thermal shift of the peaks of PVD- and CVD-SiN_x_ rings are within 100 GHz at 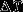 = 52 °C, while that of the Si peak moves by 100 GHz at 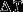 <10 °C. Considering that the ITU-T recommendation of the crosstalk is –30 dB, the minimum channel spacing of the present SiN_x_ ring should be 136 GHz according to Table 1. It is clear that our MiDex platform, non-stoichiometric SiN_x_ core and SiO_2_ cladding, is suitable to implement DWDM on a chip with the minimum channel spacing of 136 GHz. However, further stabilization is required to achieve 100 GHz or narrower spacing.

### 5.4. Athermalization

Athermalization is known to be effective to stabilize the channel wavelength, using an uppercladding material with its negative index dependence on temperature [36]. This is expressed as:


(4)
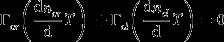



Here, Γ denotes the mode’s confinement factor of the mode, the subscript *cr* is the core, and *cl* is the cladding.

**Figure 8.**
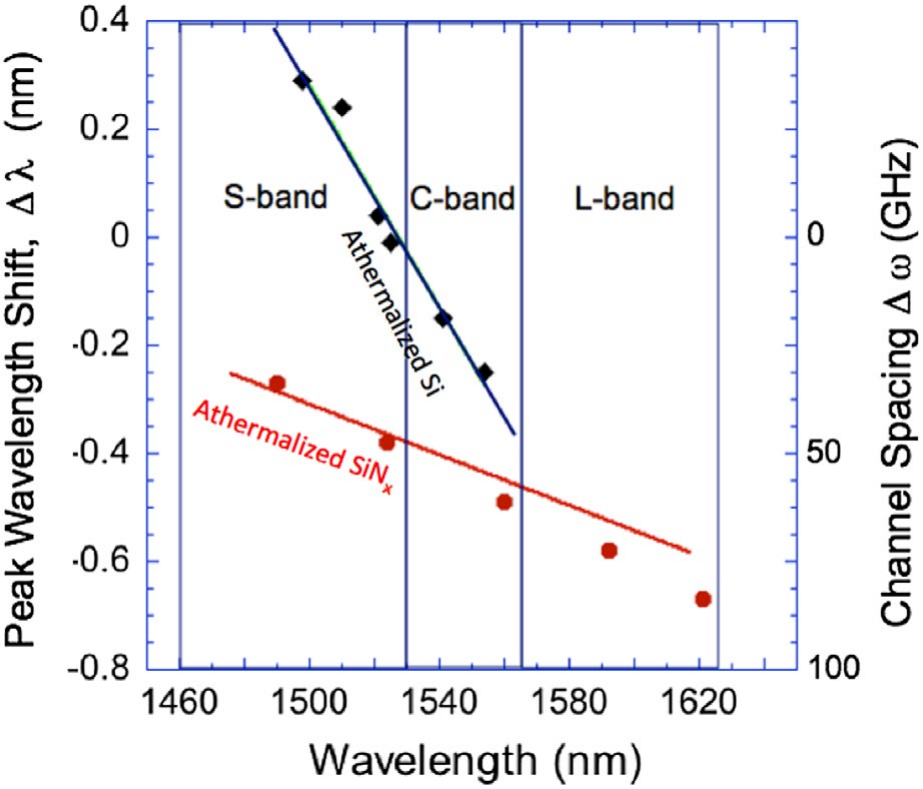
The peak wavelength shift of the PVD-SiN_x_ ring resonators with athermalization using a ‘silicone’ polymer as the upper cladding (red). The shifts were measured between 24 and 70 °C. The black and red lines are simulations. An athermalized SiN_x_ (MiDex) ring shows a smaller thermal shift than reported for an athermalized Si rings (black).[36] The athermalized SiN_x_ ring covers S-, L- and U-bands within the 50 GHz channel spacing at this temperature range, while the reported, athermalized Si rings cover C band.

The thermal peak shifts of athermalized PVD-SiN_x_ rings we measured are shown in Figure 8. Here, a polymer ‘silicone’ sold by Shin-Etsu Chemical Co (13-1, Isobe 2-chome, Annaka, Japan). Ltd is used for athermalization. We first used spectroscopic ellipsometry to measure the thermo-optic coefficient of silicone using the same heating setup we used:(5)
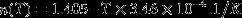



The thermal peak shifts of the SiN_x_ ring are measured at the same temperature range employed in [36], i.e. room temperature and 70 °Cin a wider wavelength range from 1490 nm to 1630 nm. The shifts calculated using Equations (4) and (5) are shown in red, which reproduces the peak shifts of the SiN_x_ ring quite well. It is demonstrated that the peak shifts of the athermalized SiN_x_ ring is within 0.4 nm, i.e. 50 GHz in the S-, C-, and L-band in this temperature range. While preserving the crosstalk of -30 dB shown in Table 1, the minimum channel spacing should be 86 GHz <100 GHz, which meets ITU-T recommendation. On the other hand, the thermal peak shift of athermalized Si rings reported using TM mode [36] is also plotted in Figure 8, indicating that there is no thermal shift at 1530 nm between room temperature and 70 °C. However, it is hard to keep the athermalization effect in a wider wavelength range as well as for TE modes since the mode profile in the Si waveguide is sensitive to its wavelength and waveguide cross section.

It is concluded that athermalization is an excellent way to further stabilize the MiDex channel wavelength against thermal fluctuation on a chip as well. Indeed, athermalized SiN_x_ rings with the silicone polymer demonstrate its great potential to lock the channel wavelength in a wider wavelength range than athermalized Si rings. Further optimization including both TE and TM athermalization will help MUX/DEMUX implemented on a DWDM chip.

## 6. Discussion

### 6.1. Ultimate MiDex material candidates for DWDM

**Figure 9.**
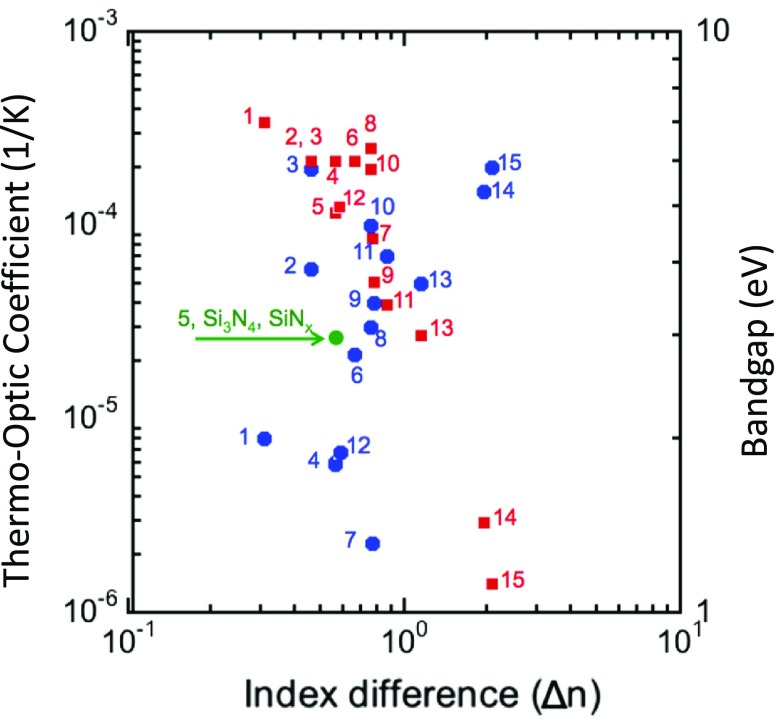
Design figure of thermo optic coefficient (blue circles) and bandgap (red squares) vs. Δn. The PVD-SiN_x_ and CVD-SiN_x_ are within the green circle of Si_3_N_4_. The following four materials, (1) Al_2_O_3_, (4) HfO_2_, (7) Ta_2_O_5_ and (12) Ga_2_O_3_, should be excellent candidates because of their low TO coefficients < 10^−5^ 1/K and the wide bandgaps >4 eV.

Further stabilization of the channel wavelength can be done by choosing the other MiDex materials. Figure 9 shows more MiDex materials [37–42] as also shown in Table 2. Among them, the following four materials: (1) Al_2_O_3_, (4) HfO_2_, (7) Ta_2_O_5_ and (12) Ga_2_O_3_ should be excellent candidates because of their low TO coefficient <10^−5^ 1/K and the bandgap >4 eV. It is interesting to note that Ta_2_O_5_ has the smallest TO coefficient, 0.23 × 10^−5^ 1/K, which is even smaller than that of SiO_2_. Δ*n* of Ta_2_O_5_ core and SiO_2_ cladding is 0.76, allowing 4 μm as the minimum bending radius as in Figure 4(a). Since the TO coefficient is one order of magnitude smaller than that of SiN_x_, simple extrapolation of the present SiN_x_ results to Ta_2_O_5_ suggests that the thermal shift of the Ta_2_O_5_ ring resonance would be only a few pm K^–1^ at 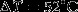. In addition, Δ*n* of Al_2_O_3_ is 0.32, which requires 100 μm of the minimum bending radius, while Ga_2_O_3_ has Δ*n* of ~0.5, allowing ~4 μm of the minimum bending radius. Therefore, a strong demand exists to fabricate the waveguide structures of the ring of these materials and their alloys and to measure transmission losses as well as mechanical (stress) and thermal stability. In addition to these materials, silicon oxynitride (SiO_x_N_y_) is proposed to tune the index by choosing the alloy composition [26]. It should also be noted that the TO coefficient of the alloy can be controlled in a range possibly between 0.24 × 10^−4^ of SiN_x_ (the present work), and 0.85 × 10^−5^ of SiO_2_ [14]. It is important to learn how much we can reduce the TO coefficient in this alloying, since the alloy has already been applied to the waveguides using CMOS technology.

**Table 2.** The MiDex materials characteristics.

Number in Figure 9	Material	Refractive index	Thermo-optic coefficient × 10^−5^ (1/K)	Bandgap (eV) at 1.55 μm
1	Al_2_O_3_	1.76	0.8	7
2	ZrSiO_4_	1.9	6	6
3	HfSiO_4_	1.9	19.7	6
4	HfO_2_	2	0.58	6
5	Si_3_N_4_, SiN_x_ (*)	2.0, 1.96 (*)	2.4, 2.8(*)	4.9
6	Y_2_O_3_	2.1	2.17	6
7	Ta_2_O_5_	2.2	0.23	4.4
8	AlN	2.19	3.0	6.3
9	LiNbO_3_	2.21	4	3.7
10	ZrO_2_	2.19	10	5.8
11	GaN	2.3	7	3.4
12	Ga_2_O_3_	1.94	0.7	5
13	SiC	2.58	5	3
14	GaAs	3.38	15	1.43
15	c-Si	3.5	20	1.12

^*^ The present PVD work.

The refractive indices were reported at near infrared around 1.55 μm for 13, 14, 15, and others are visible range at around 633 nm.

### 6.2. Monolithic EPICs on Si bulk wafers

The MiDex platform can be fabricated on any substrates including glass or polymer, in fact, any substrates. It should be noted that the recent demonstration of Ge laser diodes (LDs) on Si [43–45] clearly indicates that the MiDex platform is an enabler of ‘monolithic’ EPICs with no hybrid bonding of lasers. There have already been quite a few reports on Si photonics on Si bulk wafers and even on a CMOS chip with a high density, high performance transistors [46,47,49–53]. They were crystalline or amorphous Si waveguides fabricated on air cavities as under cladding, which were post-fabricated by etching after entire CMOS chip fabrication. Thermal fluctuation on a chip by its operation [9,10] must be cancelled by local heaters to keep the temperature of the Si rings unchanged. The challenge is the power consumption. On the other hand, only a few papers on the MiDex platform on Si bulk wafer have been reported [13,54–57]. In [55], CVD-SiN_x_ waveguides with rings were fabricated on a CMOS chip. The challenging issue is the reduction of the N–H bonds induced by the CVD process since a high temperature annealing is not applicable to CMOS circuitry under the waveguides. PVD-SiN_x_ presented in this paper should be a viable solution for the issue.

**Figure 10.**
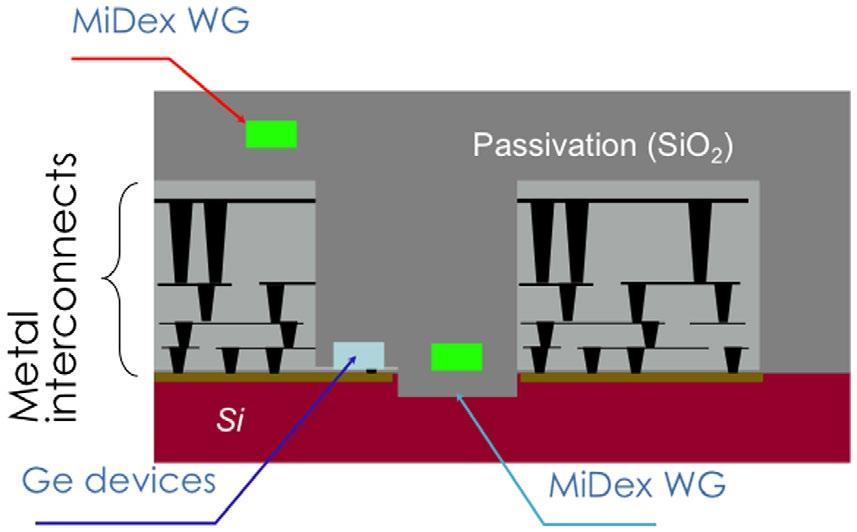
Schematics of a monolithic electronic and photonic integrated circuits on a Si bulk wafer (cross section). WG stands for waveguide.

Figure 10 illustrates a cross-sectional monolithic EPIC on a Si bulk platform. An application to a many-core chip is shown. With multilayer metal interconnection in the electronics part, Ge should be on Si next to electronics, and the MiDex waveguides can be on or between electronics parts as in the figure. There should be trenches on the Si wafer a couple of μm deep, which are filled with SiO_2_. Ge devices such as lasers, modulators, photodetectors, and MiDex waveguides could be located at the same height for a smooth coupling of the MiDex waveguide and the Ge devices and to avoid substrate coupling of the MiDex waveguides.

### 6.3. Application of MiDex Si photonics

Recently, optical data communication in vehicles has attracted strong interests because of their lower weight and faster data transmission rate as compared to existing metal cables for electrical communications. Self-driving vehicles will transfer much more information than conventional cars. Also, the ambient temperature in vehicles is relatively high. Thus, since MiDex materials are robust to temperature fluctuations, there should be a strong need for MiDex Si photonics in the automotive industry. Furthermore, in-vehicle optical communications use visible light centered at around 550 nm (2.25 eV), because it fits best to the highest transmission window of plastic optical fiber (POF), and hence cannot employ Si waveguides. However, the MiDex materials are transparent in visible range because of their bandgaps (>3 eV). For example, a Si_3_N_4_ waveguide has been used at 633 nm [16]. SiNx single-mode waveguides have also been grown by plasma-enhanced CVD for operation at 532, 780, and 900 nm with a loss below 0.5 dB cm^–1^ [56]. Meanwhile, photodetectors for in-vehicle communication do not have to be transparent and can be Si, which is available as the substrate.

In addition to the in-vehicle communication, there are many potential applications such as sensing of gas molecules in medical and environmental fields [57,58], besides existing application in e.g. data centers. Development of MiDex materials science, engineering, process and integration on Si is the key to success.

## 7. Conclusions

The mid-index contrast optics (MiDex) materials have smaller thermo-optic coefficients and wider bandgaps than Si and other high-index contrast optics (HiDex) materials do. Thus, the MiDex Si photonics platform should be robust to large thermal fluctuation on a chip and to high power transmission in terms of many wavelength channels. This opens up a new era employing the architecture of dense wavelength division multiplexing (DWDM) on a chip. To demonstrate the potentiality of the platform, we chose non-stoichiometric silicon nitride (SiN_x_) as a typical MiDex material, and physical vapor deposition (PVD) and chemical vapor deposition (CVD) methods for fabrication. It is demonstrated that the TO coefficients are 2.4 × 10^−5^ 1/K for the CVD-SiN_x_ and 2.8 × 10^−5^ 1/K for the PVD-SiN_x_. The CVD- and PVD-SiN_x_ rings have locked the peak within 100 GHz in the temperature range 24–76 °C, while they have locked within 136 GHz in the same temperature range when the crosstalk of –30 dB is considered. The athermalized SiN_x_ ring with a silicone polymer as an upper cladding further stabilizes the thermal peak shift within 86 GHz in the S, C, and L-bands in the same temperature range without the crosstalk. This meets the ITU-T protocol for DWDM. PVD-SiN_x_ needs no high temperature annealing to evacuate hydrogen, and thus is ready for the implementation in the CMOS back-end-of-line. The survey of MiDex materials indicates that Al_2_O_3_, HfO_2_, Ta_2_O_5_ and Ga_2_O_3_ should be excellent candidates for DWDM because of their low TO coefficient <10^−5^ 1/K. All the MiDex platforms including these new materials have wide bandgaps >3 eV and thus should have negligibly small optical nonlinearity at 1.55 μm. This allows DWDM implementation on electronic and photonic integrated circuit (EPIC) chips. The platform also allows manufacturing monolithic EPICs on Si bulk wafers.

Si photonics has not been an attractive field for material scientists and engineers, since EPICs can be fabricated using only CMOS materials and process technologies, which are already established for current LSIs. However, recent demand due to a significant increase in information capacity for computation and communication requires on-chip DWDM. It is now important that materials scientists and engineers use their expertise to enable development of the MiDex platform on Si.

## Disclosure statement

No potential conflict of interest was reported by the authors.

## Funding

The research results were supported by ‘R&D on optical PLL device for receiving and monitoring optical signals’, the Commissioned Research of National Institute of Information and Communications Technology (NICT), JAPAN.
